# Periconceptional Undernutrition in Sheep Affects Adult Phenotype Only in Males

**DOI:** 10.1155/2012/123610

**Published:** 2012-10-02

**Authors:** Anne L. Jaquiery, Mark H. Oliver, Maggie Honeyfield-Ross, Jane E. Harding, Frank H. Bloomfield

**Affiliations:** ^1^Liggins Institute, University of Auckland, Auckland 1023, New Zealand; ^2^National Research Centre for Growth and Development, Auckland 1023, New Zealand; ^3^Waikato Clinical School, University of Auckland, Hamilton 3240, New Zealand; ^4^Department of Paediatrics: Child and Youth Health, University of Auckland, Auckland 1023, New Zealand

## Abstract

Periconceptional undernutrition (PCUN) in sheep alters fetal growth and metabolism and postnatal growth regulation, but effects on adult body composition are unknown. We investigated the effects of PCUN on adult phenotype. Singleton lambs of ewes fed normally (N, *n* = 17) or undernourished before (UN-61-0 d, *n* = 23), before and after (UN-61-30 d, *n* = 19), or after (UN-2-30d, *n* = 17) mating (d0) were weighed at birth, 12 weeks, and intermittently to adulthood. At the age of 3-4 years, body composition was assessed by dual-emission X-ray absorptiometry followed by *postmortem* examination. Compared with N animals, male, but not female, offspring of all UN groups had greater % fat mass (all UN versus N: 9 ± 1 versus 2 ± 1%, *P* < 0.001) and perirenal fat (544 ± 36 versus 222 ± 44 g, *P* = 0.002), and proportionately smaller hearts (4.5 ± 0.1 versus 5.2 ± 0.2 g*·*kg^−1^), lungs (9.1 ± 0.2 versus 10.6 ± 0.5 g*·*kg^−1^), and adrenals (0.06 ± 0.002 versus 0.08 ± 0.003 g*·*kg^−1^). UN males also had larger testes (726 ± 21 versus 545 ± 32 g, *P* = 0.007), but UN females had smaller ovaries (2.7 ± 0.08 versus 3.4 ± 0.4 g, *P* = 0.01). Changes were independent of birth weight or postnatal growth velocity. Brief PCUN has sex-specific effects on adult phenotype, predominantly affecting males, which may contribute to adverse metabolic outcomes.

## 1. Introduction

Maternal undernutrition around the time of conception in sheep alters fetal metabolic [1] and endocrine [2] function and growth trajectory [3] but does not alter birth weight. Postnatally, growth regulation is altered [4], and changes in endocrine function become apparent with increasing age, including suppressed hypothalamic-pituitary-adrenal (HPA) [5] and leptin-insulin [6] axis function and impaired glucose tolerance [7]. One possible mechanism that might contribute to this postnatal metabolic risk after periconceptional undernutrition is altered body composition, for example, if the early nutritional insult also resulted in increased relative fat mass and/or decreased lean mass. In humans, “normal weight obesity” (increased-percentage body fat despite a normal-range body mass index) has been associated with a four fold increased risk of developing metabolic disease [8]. However, it is not clear whether increased fat mass is predominantly due to pre- or postnatal events or the interaction between them.

A number of studies suggest that rapid postnatal growth particularly after *in utero* growth restriction, occurring because of a mismatch of nutrient availability before and after birth, increases relative fat mass thereby conferring an increased risk of later metabolic disease [9–11]. However, periconceptional undernutrition in sheep alters growth regulation and trajectory without overt *in utero* growth restriction [3]. Further, the normal relationships between nutrient intake, growth factors, and weight gain in the postnatal period are disrupted in offspring of periconceptionally undernourished ewes, but these effects are not associated with increased early growth velocity [4]. 

Longitudinal experimental animal studies provide one way of determining the relative contributions of periconceptional nutritional insult, and *in utero *and postnatal growth and development to later metabolic disease risk. The aim of this study was to determine the independent effects of timing and duration of maternal undernutrition; growth during different time periods; offspring sex on later phenotype. We hypothesised that periconceptional undernutrition would result in sex-specific changes in body composition in adulthood, and that these effects would be mediated through altered postnatal growth patterns. We measured growth and body composition from birth to midadulthood in male and female singleton offspring of ewes undernourished before, both before and after, or only after conception, and carcass and organ weights at *postmortem.*

## 2. Methods

Ethical approval for the study was obtained from the University of Auckland Animal Ethics Committee. Five-year old Romney ewes were randomly allocated to a control group (N) or to one of three periconceptionally undernourished groups. All ewes received the same high-quality complete feed (Camtech Nutrition Ltd., Hamilton, New Zealand) [12]. Normally nourished controls (N) were fed a maintenance ration at 3-4% body weight per day. Ewes in the 3 undernourished groups had their feed quantity adjusted on an individual basis to achieve and maintain a weight loss of 10–15% body weight, from 61 days before mating to the time of mating (day 0) (UN-61-0), from 61 days before to 30 days after mating (UN-61-30), or from 2 days before to 30 days after mating (UN-2-30); all were fed thereafter in the same way as the N group [13]. Ewes were housed indoors in a photoperiod-controlled feedlot from 71 days before mating until two weeks after lambing. During the undernutrition period, they occupied individual pens with individual feed buckets, so that intake could be accurately recorded. Ewes were synchronised for mating using progesterone-releasing devices (CIDR-G, Pfizer, Auckland, NZ); four rams were used across all mating groups. UN ewes were kept in individual pens during the undernutrition period and then returned to group pens with the control animals until three weeks before expected lambing, when all ewes were moved to individual pens, with visual and limited olfactory contact with other ewes at all times. Labour occurred spontaneously. Lambs returned to the paddock with their ewes at two weeks of age. Only singleton offspring were included in this analysis. Male lambs were not castrated, and female lambs were never mated.

Lambs were weighed and measured weekly to 12-weeks (weaning) and then intermittently to 3-4 years of age. Growth velocity was calculated using an exponential method [14].

At 3-4 years of age, body composition was assessed by dual-emission X-ray absorptiometry (DXA, Norland XR-800, Cooper Surgical Ltd., Fort Atkinson, WI, USA) [15]. Prior to DXA, sheep were fasted overnight with free access to water. Scans were performed under sedation using an equivolume mixture of diazepam 5 mg·mL^−1^ and ketamine 100 mg·mL^−1^ intravenously. Fat and lean mass were calculated using Norland software in an area defined by the thoracic inlet proximally and the base of the tail distally, and from the animal's back superiorly to the base of the humerus and femur inferiorly, and expressed as a percentage of body weight. 

*Postmortem* examinations were carried out shortly after DXA scanning (3-4 years of age), after pentobarbitone euthanasia. Body measurements included live and eviscerated weight, measurements of linear growth (including shoulder height, hind limb length, and body length from the atlas to the base of the tail), and chest and abdominal circumferences. Organs were removed quickly after euthanasia and weighed. 

Data were analysed using StatView (“StatView for windows” SAS Institute Inc. v 5). Groups and sexes were compared using ANOVA, and relationships between growth parameters (birth weight, growth velocity between birth and weaning at 12 weeks of age, and current weight) and body composition (lean and fat mass, expressed as a percentage of body weight), assessed using regression.

## 3. Results

*Postmortem *examinations were performed on 76 animals between 3 and 4 years of age (Table 1). 

### 3.1. Growth and Body Composition

#### 3.1.1. Effect of Sex and Nutritional Group

Males were heavier overall than females at birth and in adulthood (Table 1). In males, offspring of UN-61-0 and UN-61-30 ewes had greater birth weight than offspring of N ewes (*P* = 0.02), but growth velocity to weaning (GV0-12) was greatest in the N group, so that by 12 weeks, weight was not different among groups. In females, there was no effect of nutritional group on birth weight or postnatal growth velocity. At the time of DXA scanning and *postmortem* examination, there was no difference among nutritional groups in live weight of animals of either sex. 

Adult females in all groups had greater % fat mass and lesser % lean mass than adult males (Table 1, *P* < 0.0001 for each comparison). Males in all UN groups had greater % fat mass (*P* = 0.003, all UN groups combined) and tended to have lesser % lean mass (*P* = 0.06) than male controls (N) (Table 1). They also had increased perirenal fat at *postmortem *(*P* = 0.008). In females, there was no difference among groups in adult body composition.

At *postmortem* examination, males in all UN groups had decreased shoulder height compared with N males (*P* = 0.04); males in UN-61-0 and UN-61-30 groups also had shorter body length (Table 1, *P* = 0.04). Hind limb length tended to be greater in controls (*P* = 0.09), with the difference only significant between N and UN-61-0 groups (*P* = 0.03). There were no differences among the different UN groups, or between UN and N groups, in abdominal or chest circumference. In females, there were no differences among groups in body proportions.

### 3.2. Relationships between Growth and Body Composition

Adult % fat mass was not related to birth weight in males or females of any group. 

In males, there was no relationship between adult % fat mass and body weight at 12 weeks or at the time of postmortem. However, in females, adult % fat mass was positively associated with body weight at 12 weeks (all females *R* ^2^ = 0.13, *P* = 0.04), with the effect strongest in the N group (N *R* ^2^ = 0.49, *P* = 0.02). The association between % fat mass and body weight was even stronger at the time of *postmortem* (all females *R* ^2^ = 0.43, *P* < 0.001), so that the heavier female sheep in all nutritional groups were also fatter. 

Adult % fat mass was not related to growth velocity between birth and weaning (12 weeks) in males or females from any group. 

Adult % lean mass was not related to birth weight, 12 week weight, or early growth velocity in males or females of any group, but in both sexes it was inversely associated with body weight at postmortem, with the strongest relationship in females and UN-61-30 males (all males *R* ^2^ = 0.1, *P* = 0.09; UN-61-30 males *R* ^2^ = 0.56, *P* = 0.008; all females *R* ^2^ = 0.25, *P* = 0.002). 

### 3.3. Organ Weights 

#### 3.3.1. Effect of Sex and Nutrition Group

Compared with males, females in all groups had lesser weights, both absolute and relative to body weight, for heart, lungs, kidneys, pancreas, and spleen and greater brain : body weight ratio (Table 2). Liver weight was also lower in females than in males, but liver : body weight ratio was not different between sexes. 

In males, but not females, all UN groups had lesser weight relative to body weight for heart, lungs, spleen, and adrenals (Table 2). Liver weight : body weight was decreased in the UN-61-0 group compared with the N group. 

Periconceptional undernutrition affected total gonad weight in animals of both sexes, but in opposite ways, with UN males having heavier testes and UN females lighter ovaries than N animals (nutrition x sex interaction, *P* = 0.006).

## 4. Discussion

Periconceptional undernutrition in sheep resulted in sex-specific changes in adult body proportions, body composition and organ size, with increased fat mass, decreased growth of several organs, and increased gonad size in male offspring. Body composition changes occurred within a normal weight range and were independent of birth weight and early growth velocity. Intriguingly, only gonad size was affected in female offspring, but in an opposite way to males, with females exposed to periconceptional undernutrition having smaller ovaries than controls. 

It is well established that nutrition is a powerful modulator of fetal development, and that the time around conception is crucial for the development and later function of endocrine and metabolic systems regulating weight, stress hormones, and glucose metabolism [16–18]. Periconceptional undernutrition in sheep has been associated with changes in fetal growth trajectory [19], hypothalamic-pituitary-adrenal (HPA) [2] and somatotropic [20] axis function, disordered postnatal growth regulation [4], and altered glucose-insulin [7] and HPA axis [5] function in adulthood. Here, we report that despite the relatively small effects on size at birth, postnatal growth trajectory, and final body size, periconceptional undernutrition results in significantly altered body composition in a sexually dimorphic manner. The fact that all time periods of undernutrition were associated with a similar effect on male body composition suggests a direct effect on the blastocyst or early embryo, rather than on the ovum prior to conception or via an indirect effect mediated by the altered adaptation to pregnancy seen in UN-61-0 and UN-61-30 groups [21]. 

Increased fat mass despite normal body mass index (BMI), termed “normal weight obesity” (NWO), has been increasingly recognised in humans as a risk factor for cardiovascular disease, dyslipidaemia, and the metabolic syndrome [8, 22], with some sex differences in specific risk; for example, women with NWO are more at risk of cardiovascular mortality [8]. Although the adult literature focuses on recognition of the problem and possible lifestyle interventions to improve outcomes, our data suggest that, in male sheep, increased proportional body fat is most likely a result of prenatal events. In humans, associations have been found between low birth weight (reflecting poor intrauterine growth) and increased adult fat mass, and between higher birth weight and increased lean mass in young adults [23, 24]. This would also support an antenatal influence on later body composition, but not necessarily related to a particular period of gestation. Other periconceptional events include the use of artificial reproductive technologies such as *in vitro* fertilisation (IVF) and twin conception. One study of children born after IVF found perturbed body fat distribution in childhood compared with spontaneously conceived controls [25], although another found no effect of IVF on body composition [26]. A study in sheep investigating the effect of twin conception on growth and metabolism found increased proportional fat mass in young adulthood in both males and females that were conceived as twins, even if one fetus was ablated early in gestation [15], again suggesting that events around the time of conception are crucial for normal tissue differentiation. This suggests that although increased fat mass may mediate some of the metabolic risks of *in utero* nutritional insult, it may not be causal; rather, it may be another manifestation of the developmental perturbation resulting from periconceptional undernutrition. This hypothesis is further supported by the findings of epigenetic changes after periconceptional undernutrition in fetal hypothalamic genes involved in feeding regulatory networks, including proopiomelanocortin (POMC) and glucocorticoid receptor (GR) [27].

It has been suggested that rapid postnatal growth may be the pathway by which body composition is altered, and the risk of metabolic disease thereby increased after fetal growth restriction [28]. However, we found no association between birth weight, early growth rates, and adult body composition in our study. It is interesting that the disruption of postnatal growth regulation previously demonstrated in offspring of periconceptional ewes was seen in lambs of both sexes, whereas the effects on adult body composition were seen only in males. This also suggests that a mechanism other than disrupted growth regulation or postnatal growth rate has affected adult fat mass. 

Although duration and severity of undernutrition in animal studies vary considerably in the published literature, sex specificity of metabolic outcomes is a common finding. Smith et al. found increased insulin secretion in response to a glucose load at 10 weeks of age in male offspring of ewes actively losing weight at the time of conception [29], whereas female offspring were not different from controls. In contrast, we have previously reported greater impairment of glucose tolerance in female offspring after PCUN than in males [7]. In Merino D'Arles sheep, a “hardy” breed hypothesised to be less affected by undernutrition, male offspring of ewes fed 50% of their dietary requirements before and after mating had lower plasma leptin concentrations at birth than controls, whereas females had higher leptin concentrations than controls at 4 months of age [6]. We have also reported clear sex-specific differences in HPA function over time in a subset of the cohort described in this study, with onset of adrenal ACTH resistance in males much earlier than in females [5], in contrast to the study by Smith et al., in which there was no effect of maternal weight loss around conception on offspring HPA axis prior to weaning [29]. Better understanding of the signals in embryonic life that determine sex-related growth differences may direct further study in this area. The observed widespread effect on multiple organ weights in males also suggests a global, sex-specific effect in the embryonic stage of development. 

One cannot assume a direct relationship between phenotype and organ function. Adrenal size and function have been investigated in a number of species under different stressors, but the relationship between them, particularly in a basal state, is unclear and may differ between species and under different conditions [30, 31]. We found little difference between nutritional groups in pancreatic weight, despite several studies demonstrating an influence of fetal nutrition on glucose metabolism; however, the endocrine components of the pancreas comprise only a small proportion of total pancreatic mass.

The gonadal findings are interesting, and particularly the opposite effects of maternal undernutrition on gonad size in males and females. It has been suggested that an adverse *in utero* environment leads to prioritising of investment into early growth and reproductive capacity, despite later “trade offs” in size and function of other organs [32], but males and females may be affected differently. Lifelong reproductive capacity is decreased in ewes exposed to undernutrition in late fetal life or during early postnatal life [33, 34]. Female lambs of ewes undernourished from mating until midlate pregnancy had a decreased ovulation rate compared with controls, but male reproductive function was not affected [35]. In female rats, the timing of puberty and subsequent ovarian function were found to be influenced predominantly by nutritional status *in utero*, rather than by later weight gain, with the onset of puberty at a lower body weight and lower plasma preoestrus progesterone concentrations after maternal undernutrition [36]. However, ovarian size was not reported. Women born small for gestational age have been shown to have reduced uterine size and ovarian volume in adulthood [37]. We have no information about the timing of puberty, or the functional relevance of the smaller ovaries in the female offspring in our study as these ewes were not mated, so fertility was not assessed. 

In men, testicular dimensions and volume are correlated with testicular function [38, 39]. In rams, testicular size is highly correlated with sertoli cell mass [40], and testicular diameter at 150 days of postnatal age is positively associated with postpubertal testicular size and sperm output [41]. *In utero* testicular development is dependent on circulating fetal gonadotrophin concentrations, but the effect of the nutritional environment before fetal pituitary function is established is less clear. Maternal undernutrition for 50 days after mating in sheep did not affect testicular size, but was associated with increased testosterone production, advanced development of seminiferous tubules, and increased expression of steroidogenic acute regulatory protein (StAR) at day 50, implying that undernutrition may upregulate fetal testicular steroidogenesis [42]. We did not assess fertility in the rams in our study; however, the increased testicular size across all undernourished groups suggests not only sparing of but also possible enhancement of reproductive capacity in male offspring.

While serial assessments of body composition throughout the life course would have been desirable, this was not possible due to logistic constraints. The effect of ageing on fat mass might be more profound in males, and there may have been changes over time in body composition that were related to other growth parameters, for example, rapid seasonal weight changes or growth velocity in different time periods, or the timing of puberty. The attrition over time associated with large cohorts of animals over years also meant that numbers in the male control group were lower than ideal. Despite this, the magnitude of the phenotypic differences between controls and undernourished male offspring suggests a profound effect.

## 5. Conclusions

Brief undernutrition around the time of conception in sheep alters adult phenotype in male offspring, including an increase in proportional body fat. This is likely to increase the risk of metabolic dysfunction and may contribute to the observed effects of periconceptional undernutrition on endocrine and metabolic regulation. The increased gonadal size in males, despite decreased growth of several other organs, may reflect prioritising reproductive capacity in response to an adverse periconceptional environment.

## Figures and Tables

**Table 1:** Weight, body composition, and skeletal growth measurements in male and female offspring of ewes undernourished before (UN-61-0), before and after (UN-61-30), or after (UN-2-30) mating (day 0) compared with offspring of normally fed ewes (*N*).

	Male				Female			
	N	UN-61-0	UN-61-30	UN-2-30	N	UN-61-0	UN-61-30	UN-2-30
	*n* = 5	*n* = 11	*n* = 10	*n* = 12	*n* = 12	*n* = 12	*n* = 9	*n* = 5
Birth weight (kg)^##^	5.3 ± 0.6	6.4 ± 0.3*	6.7 ± 0.3*	5.6 ± 0.3	5.2 ± 0.3	5.2 ± 0.4	6.0 ± 0.2	5.2 ± 0.7
12-week weight (kg)	30 ± 3	29 ± 2	30 ± 2	28 ± 2	29 ± 2	25 ± 1	28 ± 1	26 ± 2
GV first 12 wks (g·kg^−1^·day)	22.9 ±2.1	17.6 ± 0.7*	18.0 ± 0.8*	19.9 ± 1.1	19.4 ± 0.8	19.6 ± 0.9	18.7 ± 0.7	17.9 ±1.6
10-month weight (kg)	57 ± 5	55 ± 5	55 ± 2	49 ± 2	53 ± 4	48 ± 3	46 ± 3	54 ± 3
PM live weight aged 3-4 years (kg)^###^	92 ± 5	95 ± 3	94 ± 3	97 ± 3	85 ± 3	83 ± 2	84 ± 2	84 ± 2
% fat mass^###^	2 ± 1	9 ± 1**	8 ± 1**	11 ± 1**	21 ± 3	20 ± 1	22 ± 2	20 ± 2
% lean mass^###^	72 ± 1	68 ± 1	68 ± 1	67 ± 1	64 ± 2	65 ± 1	62 ± 1	62 ± 2
Perirenal fat (g)^###^	222 ± 44	495 ± 37**	516 ± 93**	613 ± 51**	1135 ± 108	1104 ± 68	1199 ± 103	1079 ± 84
Shoulder height (cm)	91 ± 1	86 ± 2*	84 ± 1*	86 ± 1*	86 ± 1	85 ± 1	85 ± 2	85 ± 2
Body length (cm)	89 ± 3	83 ± 1*	78 ± 3*	84 ± 1*	82 ± 1	79 ± 1	82 ± 1	78 ± 2
Chest circumference (cm)	112 ± 4	112 ± 2	112 ± 2	112 ± 1	115 ± 3	111 ± 2	113 ± 2	112 ± 3
Abdominal circumference (cm)	122 ± 5	129 ± 2	126 ± 2	128 ± 1	127 ± 3	127 ± 2	129 ± 2	129 ± 3
Hind limb length (cm)^##^	72 ± 1	67 ± 1*	69 ± 2	69 ± 1	66 ± 1	65 ± 1	67 ± 1	66 ± 2

*P < 0.05, **P < 0.01, ***P < 0.001 for comparison with *N*.

^#^ *P* < 0.05, ^##^ *P* < 0.01, ^###^ *P* < 0.001 for difference between males and females.

**Table 2:** Absolute and relative organ weights at *post mortem* in male and female offspring of ewes undernourished before (UN-61-0), before and after (UN-61-30) or after (UN-2-30) mating compared with offspring of normally fed ewes (*N*).

	Male				Female			
	*N*	UN-61-0	UN-61-30	UN-2-30	*N*	UN-61-0	UN-61-30	UN-2-30
	*n* = 5	*n* = 11	*n* = 10	*n* = 12	*n* = 12	*n* = 12	*n* = 9	*n* = 5
Heart wgt (g)^###^	474 ± 23	419 ± 17*	431 ± 11	435 ± 33	393 ± 14	370 ± 16	380 ± 11	361 ± 12
Heart: body wgt (g·kg^−1^)	5.2 ± 0.2	4.4 ± 0.2**	4.5 ± 0.1**	4.5 ± 0.1**	4.6 ± 0.2	4.4 ± 0.1	4.5 ± 0.1	4.3 ± 0.1
Lung weight^###^	965 ± 46	894 ± 28	868 ± 24	849 ± 42	722 ± 25	656 ± 29	713 ± 26	733 ± 27
Lung: body wgt (g·kg^−1^)^##^	10.6 ± 0.5	9.5 ± 0.3*	9.0 ± 0.2**	8.8 ± 0.3**	8.5 ± 0.3	8.0 ± 0.4	8.6 ± 0.4	8.8 ± 0.4
Kidney wgt (g)^###^	272 ± 24	254 ± 15	287 ± 11	256 ± 10	215 ± 10	216 ± 7	219 ± 11	210 ± 10
Kidney: body wgt (g·kg^−1^)^#^	3.0 ± 0.2	2.7 ± 0.2	3.0 ± 0.1	2.7 ± 0.1	2.5 ± 0.1	2.6 ± 0.1	2.6 ± 0.1	2.5 ± 0.1
Adrenal wgt (g)	7.0 ± 0.5	6.4 ± 0.4	5.5 ± 0.4*	5.8 ± 0.2	5.6 ± 0.3	5.8 ± 0.3	5.7 ± 0.4	5.4 ± 0.5
Adrenal: body wgt (g·kg^−1^)	0.077 ± 0.003	0.068 ± 0.005**	0.057 ± 0.004**	0.060 ± 0.003	0.067 ± 0.005	0.071 ± 0.004	0.068 ± 0.004	0.065 ± 0.005
Pancreas wgt (g)^###^	126 ± 8	118 ± 6	114 ± 7	119 ± 7	97 ± 6	91 ± 5	95 ± 6	99 ± 4
Pancreas: body wgt (g·kg^−1^)^#^	1.4 ± 0.1	1.2 ± 0.05	1.2 ± 0.1	1.2 ± 0.1	1.2 ± 0.1	1.1 ± 0.05	1.1 ± 0.1	1.2 ± 0.05
Liver wgt (g)^###^	1718 ± 173	1307 ± 179	1566 ± 33	1600 ± 72	1246 ± 44	1233 ± 48	1320 ± 45	1328 ± 54
Liver: body wgt (g·kg^−1^)	18.6 ± 1.0	13.4 ± 1.7^∗∗@^	16.3 ± 0.4	16.5 ± 0.6	14.6 ± 0.5	15.0 ± 0.6	15.8 ± 0.5	15.8 ± 0.3
Spleen wgt (g)^#^	295 ± 55	369 ± 36	392 ± 27	383 ± 46	493 ± 58	445 ± 57	449 ± 45	391 ± 93
Spleen: body wgt (g·kg^−1^)^###^	3.2 ± 0.5	3.9 ± 0.3	4.1 ± 0.3	4.0 ± 0.4	5.7 ± 0.6	5.4 ± 0.7	5.4 ± 0.5	4.6 ± 1.0
Brain	110 ± 9	107 ± 2	109 ± 4	109 ± 2	105 ± 2	106 ± 2	104 ± 3	102 ± 2
Brain: body wgt (g·kg^−1^)^###^	1.2 ± 0.06	1.1 ± 0.03	1.1 ± 0.03	1.1 ± 0.02	1.2 ± 0.06	1.3 ± 0.05	1.2 ± 0.05	1.2 ± 0.04
Gonad wgt (g)	545 ± 32	735 ± 46**	676 ± 37	759 ± 25**	3.4 ± 0.3	2.7 ± 0.1**	2.9 ± 0.1	2.3 ± 0.1**
Gonad: body wgt (g·kg^−1^)	6.1 ± 0.7	7.9 ± 0.6*	7.1 ± 0.4	7.9 ± 1.0*	0.040 ± 0.004	0.033 ± 0.002	0.034 ± 0.001	0.028 ± 0.001*

Wgt: weight

*P < 0.05, **P < 0.01, ***P < 0.001 for comparison with *N*

^@^ *P* < 0.05 for comparison with UN-2-30

^#^ *P* < 0.05, ^##^ *P* < 0.01, ^###^ *P* < 0.001 for difference between males and females.
